# Herbal Medicine for the Treatment of Obesity: An Overview of Scientific Evidence from 2007 to 2017

**DOI:** 10.1155/2017/8943059

**Published:** 2017-09-25

**Authors:** Yanfei Liu, Mingyue Sun, Hezhi Yao, Yue Liu, Rui Gao

**Affiliations:** ^1^Graduate School of Beijing University of Chinese Medicine, Beijing 100029, China; ^2^Institute of Clinical Pharmacology, Xiyuan Hospital of China Academy of Chinese Medical Sciences, Beijing 100091, China; ^3^Cardiovascular Diseases Center, Xiyuan Hospital of China Academy of Chinese Medical Sciences, Beijing 100091, China

## Abstract

Obesity is a very common global health problem, and it is known to be linked to cardiovascular and cerebrovascular diseases. Western medical treatments for obesity have many drawbacks, including effects on monoamine neurotransmitters and the potential for drug abuse and dependency. The safety of these medications requires improvement. Herbal medicine has been used for treatment of disease for more than 2000 years, and it has proven efficacy. Many studies have confirmed that herbal medicine is effective in the treatment of obesity, but the mechanisms are not clear. This article will discuss the possible effects and mechanisms of herbal medicine treatments for obesity that have been reported in the past decade.

## 1. Introduction

Obesity is a metabolic disorder characterized by an excess accumulation of fat in the body due to energy intake exceeding energy expenditure [1]. Obesity is an increasingly common phenomenon all over the world. Body mass index (BMI) is the most commonly used measure to evaluate the degree of obesity. In 2016, the AACE (the American Association of Clinical Endocrinologists) released new diagnostic criteria of obesity based on BMI combined with obesity-related complications (see Table 1) [2]. The latest study, which analyzed data from 68.5 million persons between 1980 and 2015, found that a total of 107.7 million children and 603.7 million adults were obese in 2015 [3]. Obesity has become a worldwide epidemic, and the trend is becoming increasingly serious. Obesity is an independent risk factor for metabolic syndrome; major medical problems associated with the development of hypertension, type 2 diabetes (T2DM), dyslipidemia, sleep apnea, and respiratory disorders; and ultimately life-threatening cardiovascular disease (CVD), stroke, and certain types of cancer [4–6].

The number of obese patients is increasing globally [7]. Reducing body weight by lifestyle alteration is advisable, but sometimes drug intervention is necessary [8]. Obesity drugs can be divided into five categories: central appetite suppressants, digestion and absorption blockers, metabolic promoters, obesity gene product inhibitors, and other drugs for the treatment of obesity [9]. However, the weight loss drugs prescribed in conventional medicine induce many adverse reactions, primarily effecting monoamine neurotransmitters, and causing drug abuse or dependence [10]. For example, sibutramine has been reported to commonly cause adverse events, including dry mouth, insomnia, anorexia, constipation, formation of thrombi, and neurological symptoms [11, 12]. Surgery is commonly used in morbidly obese patients (BMI ≥ 40 kg/m^2^) or in patients with comorbidities, such as hypertension, diabetes, and obstructive sleep apnea [13]. Common surgical complications include infection, postoperative anastomotic fistula, deep vein thrombosis, and long-term complications such as anemia and malnutrition [14, 15]. Given the dangers of obesity and the shortcomings of western medicine, alternative treatments should be further investigated. This article examines the potential role of herbal medicines in the treatment of obesity and summarizes the scientific evidence reported from 2007 to 2017.

## 2. Methods

The PubMed and Web of Science were searched for studies published from 2007 to 2017 on humans or animals. The search terms were “obesity,” “obese,” or “antiobesity” and “herbal medicine,” “plant,” “plant medicine,” or “Chinese medicine” without narrowing or limiting search items. Relevant publications with available abstracts and titles were reviewed by two reviewers.

The Clinical Trials (https://clinicaltrials.gov/) and Chinese Clinical Trial Registry (http://www.chictr.org.cn/) databases were searched for registered clinical trials of herbal medicine and obesity. The search terms were “obesity” or “obese” and “herbal medicine,” “plant,” “plant medicine,” or “Chinese medicine.”

## 3. Results

### 3.1. The Role of Herbal Medicine in Treating Obesity: Evidence from Human Studies

Eighteen randomized controlled trials (RCTs) (sample size > 50 cases) [16–33] published from 2007 to 2017 were included. Studies of herbal medicine interventions for obesity that had no obvious effects were excluded. The contents of the included 18 published RCTs are shown in Table 2. Analysis of these studies found that the maximum number of subjects was only 182, and the sample size is small. The age of the subjects ranged from 18 to 79 years. The studies were performed in many different populations. Eleven studies [16–18, 20, 21, 23, 25, 28–30, 33] mentioned complications, including hypertension, impaired glucose tolerance, spleen hypofunction, excessive sweating, nonalcoholic fatty liver disease, hyperlipidemia, and metabolic syndrome. Of the 18 studies, 6 were completed by Chinese researchers, and the remaining 12 were from Japan, Australia, Canada, USA, Russia, France, Indonesia, Korea, Indian, Thailand, and Italy. Thus, herbal medicine interventions for obesity are being studied in more countries than China. The outcome of each study varied and could be roughly divided into the following categories: (1) change in body weight: a significant decrease in body weight occurred following treatment with xin-ju-xiao-gao-fang (XJXGF, compound of rhubarb,* Coptis*, semen cassiae, and* Citrus aurantium*), yellow pea fiber, bofu-tsusho-san (compound of Radix Platycodi, Gypsum Fibrosum, talcum, Paeoniae, Scutellariae, and Glycyrrhizae), RCM-104 (compound of* Camellia sinensis*, flos sophorae, and semen cassiae), pistachio, Satiereal®, Monoselect Camellia (containing green tea extract: GreenSelect® Phytosome®), or* Nigella sativa*; (2) BMI: a significant decrease in body fat occurred following treatment with xin-ju-xiao-gao-fang, bofu-tsusho-san, RCM-104, Linggui Zhugan Decoction (compound of poria, Macrocephalae, Radix Glycyrrhizae, Ramulus Cinnamomi, and Radix Atractylodis), Pu'er tea, pistachio, or Monoselect Camellia; (3) waist or hip circumference: there was a significant decrease in waist or hip circumferences treat with the following herbal medicine from six studies: xin-ju-xiao-gao-fang, Pu'er tea, Satiereal, Catechin enriched green tea, West African Plant (*Irvingia gabonensis*), and* Cissus quadrangularis* (*Irvingia gabonensis*); (4) food intake: two studies, of RCM-104 and yellow pea fiber, referred to the influence of traditional Chinese medicine on food intake, but data were not provided; (5) other effects: homeostatic model assessment-insulin resistance (HOMA-IR), homeostatic model assessment-*β* cell function (HOMA-*β*), glycated hemoglobin (HbA1c), blood pressure (BP), quality of life, fasting insulin (FINS), and fasting plasma glucose (FPG) were detected in these trials; (6) evaluating these eighteen clinical studies based on Jadad score: it was found that the overall quality of these clinical studies is low. Of three studies, the Jadad score was 4, and the remaining studies scored below 4 scores. We found 16 registered clinical trials (see Table 3) from https://clinicaltrials.gov/ and http://www.chictr.org.cn/, and the recruiting locations vary from China and Korea to United States and Portugal, which will provide greater scientific insight into the treatment of obesity by herbal medicine all over the world.

### 3.2. The Role of Herbal Medicine in Treating Obesity: Evidence from Animal Studies

**Figure 1:**
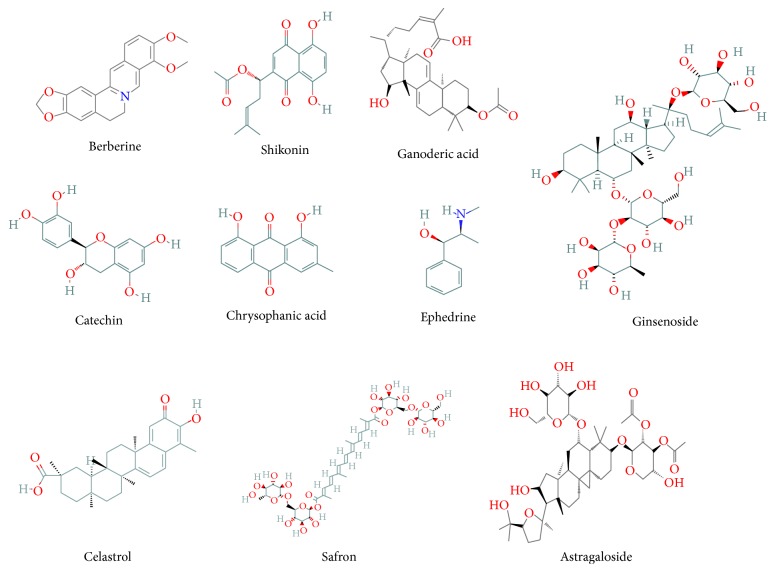
Molecular structures of the compounds described in this review.

In this section, we will summarize the known effects and mechanisms of action of single herbs and their components or extracts in animal models of obesity (see Table 4 and Figure 1).

#### 3.2.1. Rhizoma Coptidis (Huang Lian)

Rhizoma coptidis is derived from the root of* Coptis chinensis* Franch.,* Coptis deltoidea* C. Y. Cheng et Hsiao, or* Coptis teeta* Wall [55]. Its main components include alkaloids and lignans. Among the alkaloids, berberine is a main active component of Rhizoma coptidis [34]. The studies found that Rhizoma coptidis can reduce weight, lower lipids [56], reduce lipid synthesis [57], and inhibit adipogenesis [58]. Xie et al. [35] found that Rhizoma coptidis (RC) (200 mg/kg) and berberine (200 mg/kg) significantly lowered body and visceral adipose weight, reduced blood glucose and lipid levels, and decreased degradation of dietary polysaccharides in high-fat diet (HFD) mice. Both the ex vivo and* in vitro* trials confirmed that RC and berberine can regulate gut microbes to reduce weight. The antiobesity mechanisms of RC and berberine involve decreasing degradation of dietary polysaccharides, lowering caloric intake, and systemically activating fasting-induced adipose factor (FIAF) protein and expression of genes related to mitochondrial energy metabolism. Zhang et al. [59] found that when 3T3-L1 preadipocytes were cultured with various concentrations of berberine (0, 0.5, 1, 5, or 10 *μ*M) for 7 days, berberine inhibited their differentiation. Significant inhibition of intracellular lipid accumulation was observed when 3T3-L1-derived adipocytes were exposed to berberine on days 3–5 and days 5–7, and this effect was marked at 5 *μ*M berberine. The authors concluded that berberine suppresses adipocyte differentiation mainly by suppressing cAMP response element-binding protein (CREB) activity, which leads to a decrease in CCAAT/enhancer-binding protein beta- (C/EBP*β*-) triggered transcriptional cascades.

#### 3.2.2. *Panax ginseng* C. A. Mey (Ren Shen)

Ren Shen is derived from the dried root and rhizome of* Panax ginseng* C. A. Mey. (Araliaceae) [55]. Ginseng saponins and polysaccharides are the main active components of* Panax ginseng* C. A. Mey [60]. Ginseng saponins can be subdivided based on structure into Rb1, Rb2, Rc, Rd, Re, and Rl [36, 37].* Panax ginseng* C. A. Mey can reduce body weight [61], attenuate fat accumulation [62], suppress lipid accumulation and reactive oxygen species (ROS) production [63], and improve insulin resistance [64]. Li et al. [65] found that administration of ginseng (0.5 g/kg diet) to HFD-induced obese mice for 15 weeks significantly decreased body fat mass gain, improved glucose tolerance and insulin sensitivity, and prevented hypertension. Koh et al. [63] investigated the treatment of 3T3-L1 cells with Rg1 (0, 25, 50, 100, and 200 *μ*M). They observed that administration of 100 *μ*M Rg1 for 24 h greatly reduced lipid accumulation and ROS production; treatment with 100 *μ*M Rg1 in the early stages of 3T3-L1 differentiation (days 0–2) significantly decreased adipocyte formation. Rg1 reduces lipid accumulation and ROS production via the activation of C/EBP-homologous protein 10 (CHOP10), which attenuates fat accumulation and downregulates protein levels of NADPH oxidase 4 (NOX4). Lin et al. [66] found that when daily injections of 20 mg/kg Rb1 were administered to diet-induced obese mice for 3 weeks, body weight, food intake, blood glucose, and lipid levels decreased significantly. The ginsenoside, Rb1, may treat obesity by modifying the serum content and mRNA expressions of neuropeptide Y (NPY), NPY Y2 receptor, and peptide YY (PYY).

#### 3.2.3. Radix Lithospermi (Zicao)

Radix Lithospermi is derived from the root of* Arnebia euchroma* (Royle) Johnst.,* Lithospermum erythrorhizon* Sieb. et Zucc, or* Arnebia guttata* Bunge [55]. Studies have shown that Radix Lithospermi can reduce weight, inhibit lipid accumulation, induce lipolysis, and regulate lipid metabolism. The main active ingredients of Radix Lithospermi are shikonin and acetylshikonin [38, 39, 67, 68]. Su et al. [40] found that intragastric administration of 100, 300, or 900 mg/kg acetylshikonin extract for 6 weeks in obese rats significantly decreased weight, serum free fatty acid (FFA), and serum triglyceride (TG) levels. Acetylshikonin is effective in the treatment of obesity by suppressing the expression of adipogenic differentiation transcription factors and adipocyte-specific proteins, and by increasing the activity of cAMP-dependent protein kinase (PKA) and phosphorylation of hormone-sensitive lipase (HSL). Su et al. [41] found that oral gavage of 540 mg/kg/day of acetylshikonin for 8 weeks in db/db mice significantly decreased body weight, food efficiency ratio, serum TG, and FFA levels. The mechanism of acetylshikonin activity in the treatment of obesity and nonalcoholic fatty liver disease involves the regulation of lipid metabolism and anti-inflammatory effects. Bettaieb et al. [42] found that administration of shikonin (2 mg/kg/day) to HFD mice for 5 days at an injected volume of 1% of their body weight could improve glucose tolerance and decrease body weight, adiposity, and hepatic dyslipidemia over 18 weeks. Shikonin acts by enhancing hepatic insulin signaling, increasing tyrosine phosphorylation of the insulin receptor, and enhancing downstream signaling.

#### 3.2.4. *Ephedra sinica* Stapf. (Ma Huang)

The dried rhizome of* Ephedra sinica *Stapf. is used as the main ingredient of the herbal medicine, Ma Huang [69]. It has been used in recent years to treat obesity [43]. Other species that are used include* Ephedra intermedia* Schrenk et C. A. Mey. and* Ephedra equisetina* Bge [55].* Ephedra sinica* Stapf. can modulate gut microbiota, reduce weight, and improve glucose intolerance. Song et al. [70] found that oral gavage of 5%* Ephedra* and 0.5% acarbose for 6 weeks in HFD-fed mice could reduce weight gain and epididymal fat accumulation, decrease fasting blood glucose, and improve lipid profiles and glucose intolerance. The main mechanism of* Ephedra sinica*'s ability to reduce obesity and hyperglycemia involves increasing peroxisome proliferator-activated receptor alpha (PPAR-*α*) and adiponectin activity and reducing tumor necrosis factor-alpha (TNF-*α*) activity. The study published by Wang et al. [44] showed that administration of* Ephedra sinica* to HFD-induced obese rats by oral gavage over three weeks led to significant loss of body weight, epididymal fat, and perirenal fat, but no remarkable changes were observed in abdominal fat, liver weights, cecum weights, or food efficiency ratios.

#### 3.2.5. *Rheum palmatum* L. (Da-Huang)

Da-Huang is derived from the dried root and rhizome of* Rheum palmatum* L.,* Rheum tanguticum* Maxim. ex Balf, or* Rheum officinale* Baill [55]. Emodin and chrysophanic acid are the active compounds of* Rheum palmatum* L. [71]. Lim et al. [45] found that, following administration of chrysophanic acid (5 mg/kg/day) for 16 weeks to HFD mice, body weight, food intake, total cholesterol, low density lipoprotein (LDL) cholesterol, TG, and blood glucose decreased. In* in vitro* experiments, cells were cultured in medium containing chrysophanic acid for 48 h, and the results suggested that chrysophanic acid could suppresses lipid accumulation and downregulate adipogenic factors. Chrysophanic acid can ameliorate obesity by activating 5′-AMP-activated protein kinase alpha (the catalytic subunit of AMPK) to control the adipogenic and thermogenic pathway. Li et al. [46] found that administration of emodin (80 mg/kg/day) for 6 weeks to HFD-induced obese mice reduced body weights and fasting blood glucose levels, while improving insulin intolerance and serum and hepatic lipid levels. Emodin likely exerts its antiobesity effect by regulating the sterol regulatory element-binding protein (SREBP) pathway.

#### 3.2.6. Green Tea (Lvcha)

Green tea, one of the most popular teas in China, contains tea polyphenols, catechins, caffeine, and amino acids; it is frequently used to ensure weight loss [47]. Green tea induces weight loss in a variety of ways, such as activating the nuclear factor erythroid-2-related-factor-2 (Nrf2) pathway [72], upregulation of neprilysin [73], prevention of gut dysbiosis [74], regulating metabolic balance in the body, inhibiting fat accumulation and cholesterol synthesis, and reducing abdominal fat. Choi et al. [48] found that administration of an HFD plus 0.25% (w/w) green tea extract for 12 weeks in diet-induced obesity (DIO) mice ameliorated obesity, hepatic steatosis, dyslipidemia, and insulin resistance. Green tea extract contributed to the regulation of systemic metabolic homeostasis via transcriptional responses to lipid, glucose, and amino acid metabolism. Zhu et al. [75] treated 3T3-L1 cells with catechins and caffeine in various concentrations and combinations for 8 or 12 days. Combination therapy with catechins and caffeine markedly reduced intracellular fat accumulation by regulating the gene and protein expression levels of lipid metabolism-related enzymes. Yamashita et al. [76] found that, following supplementation with green tea extract powder and eriodictyol for 8 weeks, body weight, food intake, cholesterol levels, and LDL levels were decreased, accompanied by the suppression of two kinds of cholesterol synthesis enzymes, 3-hydroxy-3-methylglutaryl-coenzyme A reductase (HMGCR), and 3-hydroxy-3-methylglutaryl-coenzyme A synthase (HMGCS).

#### 3.2.7. *Astragalus membranaceus* (Fisch.) Bunge (Huang Qi or Radix Astragali)

Huang qi is derived from the dried root of* Astragalus membranaceus* (Fisch.) Bunge var.* mongholicus* or* Astragalus membranaceus* (Fisch.) Bunge [55]. The main active components of* Astragalus membranaceus* (Fisch.) Bunge are astragaloside, campanulin, ononin, kaempferol, and astragalus polysaccharides [77]. Xu et al. [49] found that when cells were incubated with isoastragaloside I (HQ1) and astragalosides II (HQ2), extracts of Radix Astragali, for 72 h, insulin resistance, and glucose intolerance were improved. Oral gavage with HQ1 and HQ2 (50 mg of each compound/kg body weight, twice a day) for 6 weeks in db/db mice increased serum levels of total adiponectin, possibly via activation of AMPK. The study published by Hoo et al. [78] suggested that daily oral gavage with Radix Astragali (2 g/kg/day) in db/db diabetic mice for 12 weeks reduces body weight and food intake and alleviates glucose intolerance/insulin resistance. The main mechanism may be the suppression of inflammatory pathways.

#### 3.2.8. *Carthamus tinctorius* L. (Hong Hua)

*Carthamus tinctorius* L. is derived from the dried flower of* Crocus sativus* L. The main active component of* Carthamus tinctorius* L. is saffron [50]. The study published by Zhu et al. [52] showed that HFD-induced obese male ICR mice, intraperitoneally injected with safflower yellow (120 mg/kg) daily for eight weeks, had significant reductions in body fat mass, fasting blood glucose, and improvements in insulin sensitivity. A possible mechanism is the promotion of the browning of subcutaneous white adipose tissue (WAT) and activating the insulin receptor substrate 1/Akt/glycogen synthase kinase 3*β* pathway in visceral WAT. Mashmoul et al. [79] used saffron (dried stigma of* Crocus sativus* L. flowers) to treat obesity-related fatty liver. They were administered saffron extract and crocin at concentrations of 40 and 80 mg/kg/day for 8 weeks in HFD-induced obese rats. Levels of liver enzymes, relative liver weights, and food intake were decreased. Saffron had a curative effect in the treatment of obesity-related fatty liver disease, but more definitive evidence of the protective effects of saffron and crocin needs to be found.

#### 3.2.9. *Ganoderma lucidum* (Leyss. ex Fr.) Karst. (Lingzhi)

Lingzhi is derived from the dried fruiting body of* Ganoderma lucidum* (Leyss. ex Fr.) [55]. Chang et al. [80] found that daily treatments for 2 months with 100 *μ*L of the water extract of* Ganoderma lucidum* mycelium at 2, 4, or 8% (w/v) by intragastric gavage in obese mice decreased weight gain and fat accumulation and decreased proinflammatory cytokine expression in the liver and adipose tissues in a dose-dependent manner. The 8% water extract of* Ganoderma lucidum* mycelium was the most effective treatment for modulating gut microbiota. The results indicate that* Ganoderma lucidum* reduces obesity in mice by modulating the composition of the gut microbiota, reducing endotoxemia, and preventing insulin resistance. Thyagarajan-Sahu et al. [81] found that treatment of 3T3-L1 preadipocytes with ReishiMax (RM, containing* Ganoderma lucidum*) (0–300 *μ*g/mL) for 9 days decreased lipid accumulation, triglyceride uptake, and glycerol accumulation in a concentration-dependent manner. RM can control adipocyte differentiation and glucose uptake, possibly via suppressed expression of the adipogenic transcription factor, PPAR-*γ*, and enzymes and proteins responsible for lipid synthesis.

#### 3.2.10. *Tripterygium wilfordii* Hook. f (Lei Gong Teng or Thunder God Vine)

Lei Gong Teng is derived from the dried roots, leaves, and flowers of* Tripterygium wilfordii* Hook. f [55]. Celastrol is the main active ingredient of* Tripterygium wilfordii* Hook. f [82]. Liu et al. [53] administered Celastrol (100 *μ*g/kg) intraperitoneally for three weeks to HFD-induced obese mice and found that Celastrol suppressed food intake, improved energy expenditure, and leads up to 45% weight loss in hyperleptinemic diet-induced obese mice by increasing leptin sensitivity. Following treatment with vehicle or Celastrol (100 *μ*g/kg) (daily, intraperitoneal) in db/db or ob/ob mice, food intake slightly decreased during the first week, and body weight, lean mass, and fat percentage were not affected by Celastrol treatment. Celastrol is a leptin sensitizer, and its main mechanism of weight reduction is relief of endoplasmic reticulum (ER) stress and increased leptin sensitivity. In another study [83], Hu et al. identified Nur77 as a critical intracellular target of Celastrol, which induces apoptosis by targeting mitochondria. Hu et al. used their findings to develop a safe and effective drug to reduce weight. Thunder god vine should be used cautiously because of its complex composition [54] and potential adverse reactions [84].

## 4. Conclusions and Perspectives

The effect and the relevant mechanisms behind how herbal medicine work as an antiobesity treatment are still controversial. During the past decade, much recent progress has been made in the study of weight loss therapy with herbal medicine. Clinical investigations of herbal medicine have been shown to be effective in the treatment of obesity, and animal experiments have begun to reveal the potential mechanisms of the various herbal medicine. However, there are some limitations as follows: (1) Obesity is associated with oxidative stress, but there have been fewer reports in this area. Flos carthami has been shown to be effective against oxidative stress and further study of oxidative stress and weight loss using safflower is warranted. (2) Some herbal treatments also show some toxicity and should be used with caution. For example, the drug composition of thunder god vine is complex, and when it is used to treat obesity, liver and kidney function should be closely monitored. There are many herbal medicines that have adverse effects if used on long-term or at the incorrect dosages, so the long-term application of herbal medicine for obesity should focus on the safety evaluation; for example, in one case [18], a skin rash was reported in the XJXGF formula group, but the rash was transient and disappeared without treatment. (3) Clinical reports indicate that herbal medicines for obesity produce few adverse reactions, and their level of safety is acceptable. However, some cases of adverse reactions have been reported, such as a case of sudden death due to the use of green tea. Therefore, the use of traditional Chinese medicines should be regulated. (4) The drug composition of herbal medicine is complex, making it difficult to determine the mechanism(s) of action, unlike in western medicine. There was also a case report [85] of a 19-year-old obese man (120 kg) who drank large amounts of green tea (15 cups per day) with a strict diet regimen, over 2 months; he lost 30 kg of body weight. However, after his usual exercise, he died of left ventricular fibrillation. His most prevalent symptoms were gastrointestinal problems, such as dyspepsia, epigastric pain, and nausea, as well as headache. Only a small number of the studies included herein have reported that the use of herbal medicine preparations caused adverse reactions. The safety of long-term use of herbal medicine needs to be further explored.

**Figure 2:**
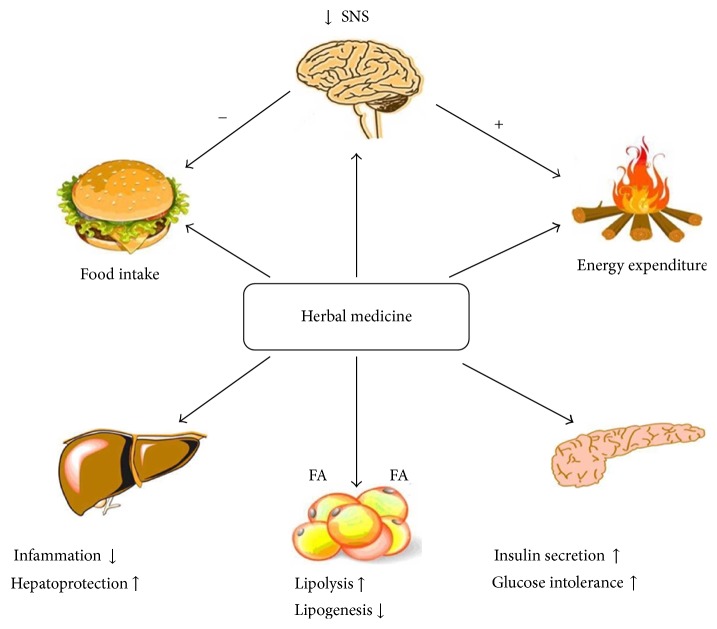
Potential mechanisms of herbal medicine for obesity. Herbal medicine such as* Celastrol*, its main mechanism of weight reduction is inhibiting endoplasmic reticulum (ER) stress and increased leptin sensitivity.* Ganoderma lucidum* mycelium decreased proinflammatory cytokine expression in the liver and adipose tissues in a dose-dependent manner;* Acetylshikonin* covers the treatment of obesity which involves the regulation of lipid metabolism and anti-inflammatory effects and decreased serum free fatty acid.* Radix Astragali* reduces body weight and food intake, and alleviates glucose intolerance/insulin resistance. SNS: sympathetic nervous system; FA: fatty acid.

Use of herbal medicine to treat obesity is currently garnering much attention. Only a small number of the active ingredients available in herbs have been identified, and if the composition of the herbs is more and more identified in the future, the target and definite mechanism of action can be determined. As mentioned above, herbal medicine has some beneficial effects on the treatment of patients with obesity and has fewer adverse effects than chemical agents; potential mechanisms of herbal medicine for obesity were presented in Figure 2. Extensive preclinical and clinical researches [86] have highlighted the pharmaceutical uses of herbal medicine as antidiabetic, antihyperlipidemic, antiobesity, anti-inflammatory, and antioxidant. In clinical practice, herbal medicines are usually used in a compound form. With the development of modern pharmacological science, it is easier to identify the active agents in herbal medicine compounds, facilitating scientific study of their effectiveness. In addition, more and more clinical trials and a standardized procedure of herbal medicine producing are needed to confirm the safety and antiobesity effect of herbal medicine and finally prevent/reduce obesity by herbal medicine consumption in human.

## Figures and Tables

**Table 1:** The new definition of obesity from the AACE^*∗*^ [2].

Diagnosis	Body mass index (BMI)	Clinical component (complications)
Overweight	≥25–29.9	No complications
Obesity stage 0	≥30	No complications
Obesity stage 1	≥25	One or more mild-to-moderate complications
Obesity stage 2	≥25	One or more severe complications

^*∗*^AACE: American Association of Clinical Endocrinologists.

**Table 2:** Published randomized controlled trials of herbal medicines for treatment of obesity in humans from 2007 to 2017.

Number	Authors/year	Targets	Age (years)	Name of herb or formula	Jadad score	Dose/duration	Groups	Main outcomes	Weight (kg) before treatment	Weight (kg) after treatment	Adverse events
(1)	Lambert et al. (2016) [16]	*n* = 53	18–70	Yellow pea fiber	4	15 g/day, 12 weeks	I: yellow pea fiber C: placebo	Body weight↓ HbAlc↓ Food intake↓ Plasma glucose↓ Satiety↑, regulating gut microbiota	92.3 ± 4.1	91.5 ± 4.0	No reports

(2)	Azushima et al, (2015) [17]	*n* = 106	20–79	Bofu-tsusho-san (Platycodi, Gypsum Fibrosum, talcum, Paeoniae, Scutellariae, Glycyrrhizae)	2	7.5 g/day, 24 weeks	I: compound C: placebo	Body weight↓ BMI↓ HbA1c↓ BP↓	82.5 ± 16.4	78.3 ± 17.9	Gastric irritation, constipation, elevation of serum hepatic enzyme level

(3)	Zhou et al. (2014) [18]	*n* = 140	18–60	Xin-ju-xiao-gao-fang (rhubarb, *Coptis*, semen cassia, *Citrus aurantium*)	3	170 mL/day, 24 weeks	I: full-dose C: low-dose	Body weight↓ BMI↓ Waist Circumference↓ Fasting insulin↓ HOMA-IR↓ HOMA-*β*↓	91.8 ± 13.4	Reduce 3.6 ± 0.5	Skin rash

(4)	Lenon et al. (2012) [19]	*n* = 117	18–60	RCM-104 (*Camellia sinensis*, flos sophorae, semen cassiae)	4	500 mg granule extract/day, 12 weeks	I: compound C: placebo	Body weight↓ BMI↓ Body fat↓ Food intake↓	99.5 ± 15.1	98 ± 15.4	Nausea, headache

(5)	Ke et al. (2012) [20]	*n* = 95	25–70	Linggui Zhugan Decoction (poria Macrocephalae, Radix Glycyrrhizae, Ramulus Cinnamomi, Radix Atractylodis)	2	Dose is unknown twice a day, 24 weeks	I: Linggui Zhugan Decoction combined with short-term very low calorie diets C: basic weight-reduction treatment	BMI↓, SBP↓ DBP↓, FPG↓ 2hPG↓, TC↓ TG↓	99.5 ± 15.1	/	Fatigue, hunger, dizziness

(6)	Chu et al. (2011) [21]	*n* = 90	18–70	Pu'er tea	3	4 cap/day, 12 weeks	I: extract C: placebo	BMI↓ Waist-hip ratio↓ TC↓ TG↓ FBG↓ PG2h↓	/	/	Diarrhea

(7)	Li et al. (2010) [22]	*n* = 59	20–65	Pistachio	3	53 g/day 12 weeks	I: pistachio C: pretzels	Body weight↓ BMI↓	86.1 ± 1.4	82.4 ± 1.6	No reports

(8)	Abidov et al. (2010) [23]	*n* = 151	/	Xanthigen (brown marine algae fucoxanthin, pomegranate seed oil)	4	2.4 mg/day, 16 weeks	I: extract C: control	Body weight↓ Body liver fat content↓	92.5 ± 1.5	88.2 ± 1.9	No adverse effects

(9)	Gout et al. (2010) [24]	*n* = 60	25–45	Satiereal, (*Crocus sativus * L. extract)	1	176.5 mg/day, 8 weeks	I: extract C: placebo	Body weight↓ BMI↓ Waist circumference↓	73.2 ± 1.1	72.2 ± 1.2	Nausea, diarrhea, reflux

(10)	Datau et al. (2010) [25]	*n* = 50	30–45	*Nigella sativa*	2	750 mg twice daily, 12 weeks	I: extract C: flour	Body weight↓ SBP↓	77.1 ± 4.9	72.6 ± 5.4	No reports

(11)	Di Pierro et al. (2009) [26]	*n* = 100	25–60	Green tea extract	1	50 mg/day 90 days	I: hypocaloric diet + extract C: hypocaloric diet	Body weight↓ BMI↓	96.1 ± 18.0	82.3 ± 15.3	No reports

(12)	Wang et al. (2009) [27]	*n* = 182	18–55	Catechin enriched green tea	2	458 mg, 468 mg, 886 mg/day, 90 days	I: extract C: Placebo	Body weight↓ Waist circumference↓ Total body fat↓	71.1 ± 11.9	69.9 ± 12.1	No adverse events

(13)	He et al. (2009) [28]	*n* = 102	18–65	Oolong tea	1	8 g/6 weeks	I: extract C: control	Body weight↓ Subcutaneous fat content↓, TC↓, TG↓	Men: 79.7 ± 6.7 Women: 70.2 ± 6.8	Women: 67.8 ± 6.7 Men: 70.2 ± 6.8	No adverse events

(14)	Ngondi et al. (2009) [29]	*n* = 102	19–50	West African Plant *(Irvingia gabonensis)*	2	150 mg/10 weeks	I: extract C: placebo	Body weight↓, body fat↓ Waist circumference↓ TC↓, LDL-C↓ Leptin levels↑	97.9 ± 9.1	85.1 ± 3.1	Headache, sleep difficulty, intestinal flatulence

(15)	Oben et al. (2008) [30]	*n* = 72	21–44	*Cissus quadrangularis*, *Irvingia gabonensis*	2	Unknown/twice daily/10 weeks	I: *Cissus quadrangularis* or *Cissus quadrangularis-Irvingia gabonensis* combination; C: placebo	Body weight↓, body fat↓, waist size↓, FBG↓, LDL-C↓ TC↓	99.8 ± 13.5	88.0 ± 3.2	Headache, lack of sleep, gas

(16)	Roongpisuthipong et al. (2007) [31]	*n* = 50	18–75	*Garcinia atroviridis*	2	1.15 grams of *Garcinia atroviridis*/day/8 weeks	I: diet + extract C: diet	Body weight↓ BMI, ↓ triceps skin fold thickness↓	69 ± 1	Reduce 2.8 ± 0.1	No adverse events

(17)	Kuriyan et al. (2007) [32]	*n* = 50	28–53	*Caralluma fimbriata*	3	1 g/60 days	I: weight loss program + extract; C: weight loss program	Hunger levels Body weight↓ BMI↓, body fat↓ energy intake↓	79.5 ± 16.9	77.2 ± 8.6	Abdominal distention, flatulence, constipation, gastritis

(18)	Oben et al. (2007) [33]	*n* = 168	19–54	*Cissus quadrangularis*	2	300, 1028 mg/8 weeks	I: two-extract formulation: CQR-300, CORE; C: placebo	Body weight↓ Body fat↓ glucose↓ HDL-C↑	118.6 ± 3.8	113.8 ± 2.5	No reports

**Table 3:** The registered clinical trials of herbal medicines for the treatment of obesity from 2007 to 2017.

Number	Trial number status	Conditions and dosage	Objectives	Interventions	Outcomes	Number of subjects (age/sex^*∗*^)	Date^*∗*^	Recruiting study locations
(1)	ChiCTR-IOR-15007587 (pending)	Obesity (/)	To evaluate the effectiveness and safety of the empirical formula—Xiere Huazhuo Formula of Chinese Medicine Professor—Ding Xueping in obesity treatment	I: Xiere Huazhuo granule; C: orlistat	1: weight, body fat distribution, blood lipid, insulin resistance HOMA-2, adipokines	48 (18–65/F-M)	December 14, 2015	China

(2)	NCT00383058 (completed)	Obesity (/)	To examine whether extract of the green tea is effective on obese women	I: the extract of green tea; C: placebo	Body mass index, body weight, glucose, cholesterol, LDL, HDL, triglyceride	100 (16–60/F)	September 29, 2009	China

(3)	NCT02605655 (completed)	Metabolic syndrome X (1 g/day for 3 months)	To determine whether the Chinese formula AMP-1915 has effect on metabolic syndrome (MS) in MS patients	I: AMP-1915 (Astragalus, Radix Puerariae, Cortex Mori); C: placebo	FBG, plasma lipid levels, plasma insulin concentration, body weight, HbA1c	60 (40–65/F-M)	April 1, 2015	China

(4)	NCT01142076 (completed)	Overweight (170 mL/day, 24 weeks)	To examine the treatment of adiposity (stagnation of QI causing phlegm retention)	I: Xinju Xiaogao Prescription; C: placebo	Waistline, BMI	140 (16–80/F-M)	March 1, 2011	China

(5)	NCT02651454 (recruiting)	Obesity (6 g, three times a day, 12 weeks)	To investigate the efficacy and safety of Daesiho-tang and Taeeumjowi-tang on Korean obese women with metabolic syndrome risk factors	I: Daesiho-tang: Jowiseungcheung-tang; C: placebo	Body weight, body fat percentage, fat mass, waist circumference, body mass index, lipid profile	120 (18–65/F)	January 5, 2016	Korea

(6)	NCT02337933 (completed)	Metabolic syndrome X (150 mg, once a day, 12 weeks)	To evaluate the effect of ursolic acid on the insulin sensitivity and metabolic syndrome	I: ursolic acid; C: placebo	Total insulin sensitivity, waist circumference, fasting glucose levels, body weight, BMI	24 (30–60/F-M)	September 1, 2015	Mexico

(7)	NCT01724099 (recruiting)	Obesity (3 times per day, 12 weeks)	To evaluate the effect of Euiiyin-tang on obese patients	I: Euiiyin-tang; C: placebo	Weight, C-reactive protein, blood pressure, blood glucose, waist/hip ratio	160 (18–65/F)	November 2, 2012	Korea

(8)	NCT02929849 (ongoing)	Obesity (300 mg, 500 mg/day)	To determine whether an herb with known alpha-glucosidase inhibitor properties (*Salacia chinensis*, SC), affecting postprandial appetite ratings and glucose indices in overweight/obese individuals	I: *Salacia chinensis*; C: placebo	Appetite ratings, glucose indices, gut hormones	59 (20–59/F-M)	August 16, 2016	United States

(9)	NCT01778257 (completed)	Obesity (mate extract (3150 mg/day), 12 weeks)	To evaluate efficacy and safety of mate extracts on decrement of body and abdominal fat in obese subjects	I: mate extract; C: placebo	Body and abdominal fat, weight, BMI, waist and hip circumference	30 (19–65/F-M)	March 1, 2012	Korea

(10)	NCT01709955 (completed)	Obesity (750 mg of Glucomannan in capsule form)	To determine if the herb, Glucomannan, is an effective nonpharmacological appetite suppressant for overweight or class I obese patients	I: Glucomannan, C: placebo	Weight	43 (21–60/F-M)	July 1, 2011	United States

(11)	NCT00502658 (completed)	Overweight, obesity (dose is unknown, 12 weeks)	To evaluate the effect of dietary supplements (shakes and supplements) and personal energy tracking device to promote and maintain healthy weight	I: dietary supplement containing vitamins, minerals, and herbs; C: dietary supplement	Body weight, biophotonic scanner	120 (18–65/F-M)	December 1, 2007	United States

(12)	NCT00823381 (completed)	Obesity, metabolic syndrome (75 mg once a day)	To evaluate the effects of the antioxidant “resveratrol” to a diet intervention (calorie restriction)	I: resveratrol, C: placebo	Insulin sensitivity, body composition, blood lipid levels	58 (35–70/F)	December 1, 2013	United States

(13)	NCT02613715 (completed)	Overweight and obesity (250 mL of blackberry juice)	To evaluate the bioavailability of blackberry juice anthocyanins in normal weight and overweight/obese adults	I: blackberry juice C: blackberry juice with 12% ethanol	Plasma concentrations of anthocyanins and anthocyanin metabolites	18 (18–40/M-F)	June 2015	Portugal

(14)	NCT01705093 (unknown)	Childhood obesity; cardiovascular disease (50 g of flavonoid-rich freeze-dried strawberry powder)	To verify if strawberry intake can lead to improvements in select measures of cardiovascular function in overweight and obese adolescent males	I: flavonoid-rich freeze-dried strawberry powder C: macronutrient- matched control powder	Vascular functionmeasured by peripheralarterial tonometry	25 (14–18/M)	August 2012	United States

(15)	NCT01138930 (unknown)	Polycystic ovary syndrome; obesity (1.5 g daily for 3 months)	To examine the effect of berberine metabolic and hormonal parameters and insulin resistance in obese patients with polycystic ovary syndrome	I: berberine; C: placebo	Body insulin action, Weight, waist/hip circumference, OGTT	120 (18–35/F)	June 7, 2010	China

(16)	NCT01471275 (unknown)	Type 2 diabetes mellitus; obesity; high triglycerides (15 g each time, twice a day, with boiled water)	Evaluate the safety and efficacy of Jiang Tang Tiao Zhi decoction in treatment of obesity with type 2 diabetes, dyslipidemia	I: Jiang Tang Tiao Zhi decoction; C: metformin	Glycosylated hemoglobin, waistline, triglycerides, liver function	450 (30–65/F-M)	November 14, 2011	China

^*∗*^If the status is completed, the date is completion date; others are registration date; F = female; M = men.

**Table 4:** The antiobesity effects of single herbs and their components or extracts in animal models.

Herb	Animal	Model	Dose/administration/time	Effects	Components	Reference
*Rhizoma coptidis*	Mice	High-fat diet-fed C57BL/6J mice	Berberine (200 mg/kg) oral gavage/6 weeks	Visceral adipose↓ Weight↓ Blood glucose↓ Lipid levels↓	Berberine	[34, 35]

*Panax ginseng *C. A. Mey	Mice	High-fat diet-fed mice	20 mg/kg/intragastric administration/3 weeks	Body weight↓ Food intake↓ Blood glucose↓ TC↓ TG↓	Ginsenoside Rg1 Ginsenoside Rb1 Ginsenoside Rg3	[36–38]

Radix Lithospermi	Rat	High-fat diet-fed db/db mice	Acetylshikonin extract (100, 300, or 900 mg/kg)/intragastric administration/6 weeks; db/db mice: acetylshikonin (540 mg/kg/day) oral/8 weeks	Body weight↓ FFA↓ TG↓ inhibited Differentiation Fat accumulation, Food intake↓	Acetylshikonin; shikonin	[39–42]

*Ephedra sinica *Stapf.	Male ICR mice	High-fat diet-fed	Diet containing 5% Ephedra/oral gavage/6 weeks	Body weight ↓ Fasting glucose↓ HDL-C↑	Ephedra	[43, 44]

*Rheum palmatum *L.	Mice	Obese mice	Mice: chrysophanic acid (5 mg/kg/day)/oral gavage/16 weeks	Body weight↓ TG↓ HDL-↑, TC↓ Food intake↓	Chrysophanic acid	[45–47]

*Green tea*	Mice	Diet-induced obese male C57BL/6J mice	0.25% (w/w) GT extract/oral gavage/12 weeks	Body weight↓ Energy intake HOMA-IR↓, TG ↓, TC↓ FFA↓	Catechin	[27, 48]

*Astragalus membranaceus *(Fisch.) Bunge	Mice	db/db diabetic mice	Radix Astragali (2 g/kg/day)/oral gavage/12 weeks	Body weight ↓ Food intake ↓ HDL-C↑	Astragalosides I Astragalosides II	[49, 50]

*Carthamus tinctorius L*	Rat	High-fat diet-induced obese rats	Saffron extract and crocin at concentrations of 40 and 80 mg/kg/day oral/8 weeks	Food intake, relative liver weight	Saffron, crocin	[51, 52]

*Ganoderma lucidum (Leyss. ex Fr.) Karst*	Mice	ob/ob mice	100 *µ*L water extract of *G. lucidum* mycelium/intragastric gavage/8 weeks	Inflammation endotoxemia ↓ Insulin resistance ↓ Regulated lipogenic gene expression.	Saffron	[53]

*Tripterygium wilfordii *Hook. f	Mice	High-fat diet-induced obese db/db or ob/ob mice	Celastrol (100 *µ*g/kg)/ intraperitoneally injection/3 weeks	HFD: food intake ↓ Energy expenditure ↑ Body weight↓ db/db or ob/ob mice: body weights, lean mass, and fat percentage were not affected	Celastrol	[54]

## Abbreviation

2hPG: 2 hours of postprandial blood glucose
BMI: Body mass index
BP: Blood pressure
BW: Body weight
CVD: Cardiovascular disease
DBP: Diastolic blood pressure
FBG: Fasting blood glucose
FFA: Free fatty acid
FINS: Fasting insulin
FPG: Fasting plasma glucose
HDL: High density lipoprotein
HFD: High-fat diet
HOMA: Homeostasis model assessment
IAF: Intraabdominal fat
IR: Insulin resistance
HbAlc: Glycated hemoglobin
OGTT: Oral glucose tolerance test
RCT: Randomized controlled trial
SBP: Systolic blood pressure
T2DM: Type 2 diabetes mellitus
TC: Total cholesterol
TG: Triglyceride
WC: Waist circumference.
